# Neutrophil Evolution and Their Diseases in Humans

**DOI:** 10.3389/fimmu.2017.01009

**Published:** 2017-08-28

**Authors:** Jennifer W. Leiding

**Affiliations:** ^1^Division of Allergy and Immunology, Department of Pediatrics, University of South Florida, Tampa, FL, United States; ^2^Johns Hopkins All Children’s Hospital, St. Petersburg, FL, United States

**Keywords:** neutrophil, neutropenia, chemotaxis, immunodeficiency, granulocytes

## Abstract

Granulocytes have been preserved and have evolved across species, developing into cells that provide one of the first lines of host defense against pathogens. In humans, neutrophils are involved in early recognition and killing of infectious pathogens. Disruption in neutrophil production, emigration, chemotaxis, and function cause a spectrum of primary immune defects characterized by host susceptibility to invasive infections.

## Introduction and Neutrophil Evolution

All vertebrate species possess leukocytes, which divide into several different highly specialized cell lineages involved in immune response and tissue repair. Leukocytes fall into several classes, including granulocytes, macrophages, and lymphocytes. Granulocytes are differentiated from other leukocytes based on their morphology, including a segmented nucleus and staining properties of their cytoplasmic granules (1). Granulocytes are classified according to the morphology of their nucleus and staining properties of their granules (1).

Cells exhibiting some phagocytic activity, termed amebocytes, are seen early in phylogeny in basic invertebrates lacking a true body cavity (celom) or vascular system. Cnidarians, one of the most basic invertebrates contain a gelatinous matrix between an ectoderm and endoderm that contains multiple amebocytes that aid in digestion, are continuously proliferating stem cells and act as phagocytes. Invertebrates that possess a body cavity and vascular system contain a third dermal tissue, the mesoderm that forms mesothelium within the celom. The mesothelial walls are the site of origin of blood progenitor cells, termed hemocytes. Four major hemocyte classifications derive from the mesothelial wall and are carried through phylogeny from invertebrates to vertebrates: prohemocytes that evolve into immature blood precursor cells, hyaline hemocytes that progress to plasmatocytes and then to monocytes, eleocytes that develop into other mesodermal derived tissues (i.e., the gastrointestinal tract), and granular hemocytes that develop into granulocytes involved in phagocytosis [reviewed in Ref. (1)].

The bone marrow is the principal hematopoietic organ of all vertebrates with the exception of fish. From the bloodstream, primitive hematopoietic stem cells (HSCs) arrive in the bone marrow in the last embryonic stages (1). Early in embryogenesis, erythrocytes initially are found within the yolk sac in the first 3 weeks of human gestation; the subsequent development of the vascular system allows blood cells to distribute to other embryonic tissues. By 6 weeks, the fetal liver is the major hematopoietic organ; the bone marrow takes over as the major site of hematopoiesis by the end of the second trimester (2). In the developing fetus, neutrophil progenitors are seen as early as the first trimester and increase in quantity nearly fourfold in the second trimester when the bone marrow becomes the major site of hematopoiesis. Circulating neutrophil counts rise abruptly and stabilize in the first 48–72 h of life (3). In preterm infants, the baseline neutrophil count is lower and there is no rise in neutrophil count in the first few days of life (4). In addition to quantitative impairments, neonatal neutrophils also exhibit many qualitative defects. Neutrophil adhesion is impaired by decreased levels of l-selectin and the β2 integrins CD18/CD11b and CD18/CD11a, which are adhesion molecules present on the surface of neutrophils and the endothelial surface and are imperative in neutrophil migration from the vasculature to sites of infection. l-selectin levels continue to decrease in the first 24–72 h, continue to be low in the first few weeks of life, and are even lower in preterm infants (5). Abnormal actin polymerization also is noted in the first few weeks of life causing a substantial decrease in directed migration *in vitro* (6). Although overall killing activity is not impaired, neonatal neutrophils have lower concentration of granular proteins (7). Despite these abnormalities, bone marrow production, neutrophil migration, and neutrophil activity mature rapidly, consistent with their role in serving as first responders to infectious and inflammatory stimuli.

**Figure 1:**
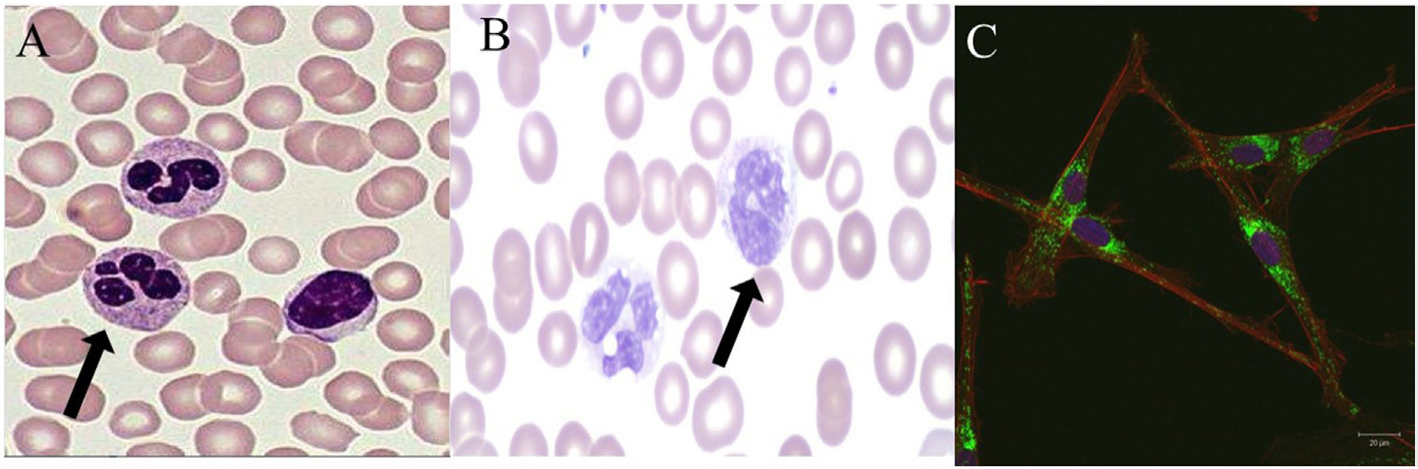
Normal and abnormal neutrophil morphology. **(A)**. Normal human neutrophils (arrow) with characteristic multi-lobed nucleus. Primary and secondary granules are visualized in the cytoplasm. **(B)**. Human neutrophils (arrow) from a patient with a mutation in *CEBP-*ε causing specific granule deficiency. Neutrophils have a characteristic bilobed nucleus and absence of specific granules in the cytoplasm. **(C)**. Human giant fused cytoplasmic granules in a patient with Chediak–Higashi syndrome. Electron microscopy at 20 µm.

**Figure 2:**
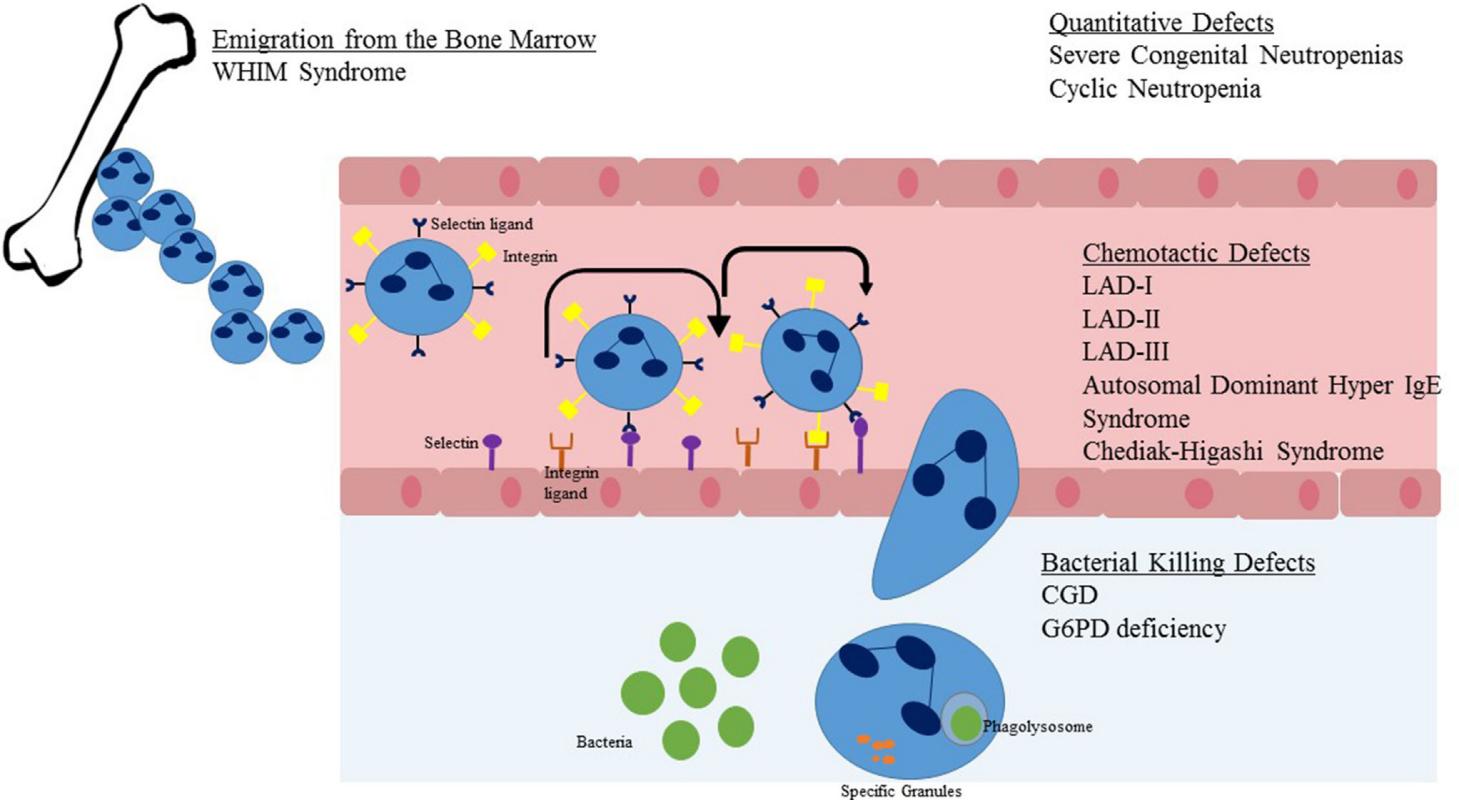
Steps in neutrophil migration from the marrow to sites of infection. Upon emigration from the bone marrow, the neutrophil travels within blood vessels. Once stimulated by chemokines or infectious pathogens in the tissue, the neutrophil begins a process of rolling adhesion to the endothelial surface and eventual migration through the endothelial wall. Once at the site of infection, the NADPH oxidase system is activated and granules are released to cause direct killing of the pathogen. Listed in text are neutrophil defects associated with the individual steps of neutrophil migration and killing.

Once developed, neutrophils are the dominant leukocyte population in humans. Neutrophils mature in the bone marrow in an orderly fashion from myeloblast to promyelocyte to myelocyte to metamyelocyte to band form and lastly the mature neutrophil. Only the latter two of these stages, the band form and mature neutrophil are present in peripheral blood. Neutrophils should have a three to four lobed nucleus and a granular cytoplasm (Figure 1A). Approximately 100 billion neutrophils enter and leave circulating blood every day (8). Neutrophils originate in the bone marrow and are released to vasculature when they have matured and are stimulated by invasive pathogens and inflammatory signals (Figure 2). Chemokines, small signaling molecules are potent chemoattractants for neutrophils to sites of infection or tissue injury. Migration toward the site of infection involves a complex multi-step process, including rolling adhesion of neutrophils on endothelial cells, firm adhesion of neutrophils, extravasation through the endothelium, and chemotactic migration. Upon migration to the site of infection, the neutrophil eliminates the invading pathogen utilizing a combination of NADPH oxidase derived reactive oxygen species, cytotoxic granule components, and neutrophil extracellular traps (8–10).

## Zebrafish Neutrophil Biology

Neutrophils are one of the first cells to respond to sites of acute infection and cell damage, playing key roles in host defense against infectious pathogens and in the development and resolution of inflammation. In order to understand the complex inflammatory process caused and resolved by neutrophils, models to investigate neutrophil biology have been developed. The short lifespan of human neutrophils is prohibitive in the investigation of neutrophil biology *in vivo* and causes *in vitro* genetic manipulation to be impractical. Because of these restrictions, a zebrafish model of neutrophil biology investigation has become widely accepted. The zebrafish neutrophil mirrors mammalian neutrophils by sharing similar morphology, and biochemical and functional features. It has a polymorphic nucleus, primary and secondary granules, and an NADPH oxidase (11, 12); multiple models of primary immunodeficiency diseases in zebrafish have been developed and studied [reviewed in Ref. (11)].

Using a zebrafish model, the mechanisms of neutrophil recruitment to sites of tissue damage have been elucidated. Damage-associated molecular patterns and chemokines recruit neutrophils effectively. Hydrogen peroxide, released by damaged tissue is one of the earliest attractants for neutrophils to sites of tissue injury. Chemokines, small signaling proteins that attract white blood cells to specific locations in the tissue first evolved ~650 million years ago in fish (13). Neutrophils respond to specific chemokine signatures from dying cells and are able to differentiate pathogen from non-infected cells. Pathogen response-like chemokines, CXCL1, CCL2, and CXCL10, are potent attractors of neutrophils leading to the development of inflammation and elimination of dying cells (14). Chemokine-induced neutrophil recruitment has been conserved across vertebrate species confirming the important process that chemoattraction plays in neutrophil recruitment (13).

Once an infection has resolved and cellular debris cleared, neutrophils must leave the site of tissue injury. High-resolution imaging of transparent zebrafish have uncovered reverse migration as a method of neutrophil resolution of inflammation. Reverse migration is a process whereby neutrophils migrate away from a site of infection or inflammation, a process regulated by pro-inflammatory cytokines (15).

When neutrophils persist in the tissue, inflammation persists and becomes chronic. Chronic neutrophil-driven inflammation has been linked to multiple autoimmune diseases and cancer progression. Neutrophils are found within many types of cancers and correlate with more aggressive disease and a poorer prognosis [reviewed in Ref. (16)]. The recruitment of neutrophils to tumor cells occurs in a similar fashion as to that of infected cells; chemokines and hydrogen peroxide produced by tumor cells attract neutrophils to tumor affected cells. Tumor-associated neutrophils are thought to play a role in cancer progression by affecting the extracellular matrix allowing for enhanced cancer cell proliferation and invasion. In addition, neutrophils also suppress anti-tumor immunity from other cell types. Targeting neutrophils has become a desirable therapeutic option for treatment of certain cancers [reviewed in Ref. (16)].

## Neutrophil Diseases in Humans

Immunodeficiency diseases afford novel insight into both normal function and pathophysiology. In terms of abnormal neutrophil function in humans, immunodeficiency that traces to abnormal neutrophil quantity or function is relatively common, occurring in approximately 20% of those with congenital primary immunodeficiency disorders. Disorders of neutrophils can be divided into four types affecting: neutrophil quantity, neutrophil granules, neutrophil chemotaxis, and neutrophil killing. This review focuses on what we have learned about the role of neutrophils in host protection from the four recognized classes of neutrophil disorders (17).

## Disorders of Neutrophil Quantity

Neutrophils live about 5 days in circulation (18) and approximately 10^11^ neutrophils (8) are made by the bone marrow each day. Neutropenia can be mild [absolute neutrophil count (ANC) 1,000–1,500 cells/μL], moderate (ANC 500–1,000 cells/μL), or severe (ANC < 500 cells/μL). Severe neutropenia is more commonly found acutely rather than chronic. However, when found, cyclic and chronic forms of severe neutropenia cause increased susceptibility to soft tissue and invasive bacterial infections. There often is a characteristic lack of pus at sites of infection (19, 20).

The genetic basis of many of the congenital forms of neutropenia have been well elucidated (Table 1). More than 50% of patients with severe congenital neutropenias (SCNs) and nearly all patients with cyclic neutropenia have autosomal dominant (AD) monoallelic mutations in *ELANE*, the gene that encodes neutrophil elastase (21, 22). Those with cyclic disease typically present in the first year of life with recurring episodes of fever and severe neutropenia in a recurring cycle usually every 21 days. During their nadir, patients are susceptible to mouth sores, soft tissue, and invasive bacterial infections. Diagnosis of cyclic neutropenia includes serial complete blood counts to capture periods of neutropenia, often requiring monitoring of the neutrophil count 2 to 3 times per week for 6–8 weeks (23). Mutations in *ELANE* also cause SCN type 1 in which neutropenia is chronic and not cyclical. ELANE is responsible for triggering an aberrant stress response in the neutrophil and when mutated leads to premature apoptosis of the neutrophil.

**Table 1:** Congenital neutropenia disorders.

Disease	Genetic defect	Inheritance	Immunologic phenotype	Other manifestations	Reference
SCN type 1	ELANE	AD	Chronic or cyclic neutropenia		(22)

SCN2	GFI1	AD	Neutropenia, lymphopenia		(24)

SCN3	HAX1	AR	Neutropenia	Neurologic impairment	(25)

SCN4	G6PC3	AR	Neutropenia Thrombocytopenia	Congenital heart defectsFacial dysmorphism increased visibility of superficial veins urogenital malformations endocrine abnormalities hearing loss skin hyperelasticity	(26)

XL congenital neutropenia	WAS	XL	Neutropenia Lymphopenia, myelodysplasia		(27)

**Congenital neutropenia and hypopigmentation disorders**					

Chediak–Higashi syndrome	LYST	AR	Neutropenia Natural killer (NK) cell dysfunction	Oculocutaneous albinismNeurologic impairment HLH	(28)

Hermansky–Pudlak syndrome type 2	AP3B1	AR	Neutropenia T and NK cell dysfunction	Oculocutaneous albinism	(29, 30)

Griscelli syndrome type 2	RAB27A	AR	Neutropenia NK cell dysfunction	Oculocutaneous albinism HLH	(31)

**Other syndromes with neutropenia as a key feature**					

Reticular dysgenesis	AK2	AR	Neutropenia Severe lymphopenia	Sensorineural hearing loss	(32)

Shwachman–Diamond syndrome	SBDS	AR	Neutropenia	Exocrine pancreatic insufficiency Skeletal dysplasia Liver and heart disease	(33)

Poikiloderma with neutropenia	C16ORF57	AR	Neutropenia	Poikiloderma, increased photosensitivity	(34)

Cartilage-Hair hypoplasia	RMRP	AR	Neutropenia T and NK cell lymphopenia	Autoimmune cytopenias Skeletal dysplasia Dwarfism	(35)

XL hyper IgM syndrome	CD40L	XL	Intermittent neutropenia Defective B cell class switching T and B cell defects		(36)

XL agammaglobulinemia	BTK	XL	Low to absent B cells Hypogammaglobulinemia		(37)

Barth syndrome	G4.5/TAZ	XL	Neutropenia	Cardioskeletal abnormalities Myopathy Growth retardation	(38)

Cohen syndrome	VPS13B/COH	AR	Intermittent neutropenia	Psychomotor retardation Skeletal dysplasia hyptonia	(39)

Pearson syndrome		Mitochondrial DNA	Neutropenia	Bone marrow failure Exocrine pancreatic insufficiency Endocrine abnormalities Neuromuscular degeneration	(40)

*AD, autosomal dominant; AR, autosomal recessive; G6PC3, glucose-6-phosphatase catalytic subunit 3; WAS, Wiskott–Aldrich syndrome; XL, X-linked; BTK, Bruton’s tyrosine kinase*.

Severe congenital neutropenia 2 is caused by mutations in *GFI1* a transcription factor that regulates normal neutrophil hematopoiesis. In addition to its effects on neutrophils, mutations in *GFI1* are associated with defects in lymphoid and myeloid cell lines (24).

Approximately 15% of SCNs are caused by autosomal recessive (AR) mutations in *HAX1* (SCN type 3). Patients with HAX1 deficiency present with marked neutropenia and may have life threatening bacterial infections as early as the newborn period. Although the exact role that HAX1 plays in neutrophil ontogeny is unknown; one suggested mechanism is that HAX1 is a major inhibitor of neutrophil apoptosis in myeloid cells and the neutropenia described in HAX1-deficient patients is due to the lack of anti-apoptotic effect (25).

Defects in glucose-6-phosphatase catalytic subunit 3 (G6PC3) cause SCN4. Patients with mutations in *G6PC3* suffer from myeloid maturation arrest leading to congenital neutropenia. They also suffer from various other congenital defects, including cardiac and urogenital defects and facial dysmorphia, increased visibility of superficial veins, inner ear hearing loss, endocrine abnormalities, or myopathy (26).

Wiskott–Aldrich syndrome (WAS) is an X-linked (XL) disorder caused by deleterious loss of function mutations in *WAS* and its cognate protein Wiskott–Aldrich syndrome protein and is characterized by susceptibility to infections, thrombocytopenia with bleeding diathesis, and eczema (41). Rare activating mutations in *WAS* cause a constitutive activation with increase in actin polymerization (27), and instead of classic WAS, these patients present with X-linked congenital neutropenia associated with myelodysplasia, lymphoid abnormalities, and increased myeloid apoptosis (42).

In contrast to SCNs in which myeloid arrest or increased apoptosis cause neutropenia, myelokathexis, or inability of neutrophils to immigrate from the bone marrow can cause severe congenital neutropenia. Warts, hypogammaglobulinemia, infections, myelokathexis syndrome, in which the clinical manifestations include neutropenia, hypogammaglobulinemia, and mild to extensive warts is an AD immunodeficiency caused by gain of function mutations in the chemokine receptor CXCR4. Stromal cell-derived growth factor-1 (SDF1, also known as CXCL12) is found in the bone marrow stroma and is the ligand for CXCR4 found on neutrophils; both are important bone marrow retention factors for neutrophils. Myelokathexis, hyperplasia with an accumulation of apoptotic neutrophils in the bone marrow and neutropenia in the periphery, is the hallmark of this disorder (43, 44).

In addition to congenital neutropenia disorders described thus far, several disorders with neutropenia and hypopigmentation also have been described (Table 1). Neutropenia may be constant in some or intermittent in others. Lastly, neutropenia leading to susceptibility to invasive bacterial infections can be a clinical manifestation in other immunodeficiency syndromes, such as XL hyper IgM syndrome (36) and XL agammaglobulinemia (37).

Patients with SCN typically present in infancy with recurrent mouth sores, pharyngitis, otitis media, respiratory infections, skin infections, and neutropenia (ANC < 200/μL). Evaluation of the bone marrow may be helpful in narrowing the differential diagnosis of congenital neutropenia. In SCN syndromes, there is a characteristic normal or decreased cellularity with early myeloid arrest at the pro-myelocte or myelocyte stages often with atypical nuclei and cytoplasmic vacuolization (45).

Treatment of SCN includes daily subcutaneous injections of recombinant granulocyte colony stimulating factor (G-CSF). Most patients with SCN respond to G-CSF; however, patients continue to be at risk for myelodysplasia, acute leukemias, and severe infections. Because of these risks and negative impact of disease on quality of life, patients with SCNs should be considered for curative therapy with HSC transplantation (20).

## Disorders of Neutrophil Chemotaxis

For efficient neutrophil killing, neutrophils must first leave the vasculature and reach a site of infection. Recruitment of neutrophils to leave the blood stream consists of three major steps: initiation of adherence of activated endothelial cells and rolling, firm attachment of neutrophils to the endothelium, and migrating of the neutrophil across the endothelial barrier (Figure 2). The initial steps occur due to interaction between P-selectin glycoprotein ligand-1 of neutrophils and P-selectin or E-selectin of endothelial cells. Firm attachment of neutrophils to the endothelium is dependent on β2 integrins (LFA-1 and Mac-1) present on the surface of neutrophils interacting with intracellular adhesion molecule-1 on endothelial cells. Final migration is triggered by local chemokines and bacterial products at the site of infection.

Defects in a number of these adhesion molecules results in clinical syndromes. Leukocyte adhesion deficiency (LAD)-I is an AR syndrome due to defects in CD18, the common β chain of the β2 integrin family. The β2 integrin is required for stable expression of three distinct β2 integrins: CD11a/CD18 (LFA-1), CD11b/CD18 (Mac-1), and CD11c/CD18 (p150,95). Patients with LAD-I typically present with early onset of soft tissue and invasive bacterial infections, delayed separation of the umbilical cord, poor wound healing, omphalitis, periodontal disease, and neutrophilia in the serum. Diagnosis of LAD-I is confirmed by absence of CD18 and the associated alpha subunits CD11a, CD11b, and CD11c or by sequencing of the β2 integrin. Treatment includes use of prophylactic antibiotics and hematopoietic stem cell transplant (HSCT) for those with a severe phenotype (46).

Leukocyte adhesion deficiency-II is a very rare AR syndrome that results from defects in the guanosine diphosphate fucose transporter gene (*SLC35C1)* leading to abnormal fucosylation on the neutrophil surface that results in defective rolling of leukocytes (46, 47). Fucosylated proteins such as sialyl Lewis X (CD15s) are ligands for endothelial selectins and are important for the early phases of adhesion. However, neutrophils are able to adhere and transmigrate *via* β2 integrins, allowing for some level of neutrophil defense against bacterial infections. Clinical manifestations include susceptibility to pyogenic infections although less severe than in LAD-I. Patients also have intellectual disability, short stature, depressed nasal bridge, microcephaly, and cortical atrophy, and the rare Bombay (hh) blood phenotype with lack of A, B, and H antigens. Absence of SLeX (CD15a) shown by analysis of peripheral leukocytes is diagnostic. Treatment includes use of prophylactic antibiotics (46, 47). Trials of fucose supplementation have been beneficial in some (47).

Leukocyte adhesion deficiency-III is a rare AR syndrome caused by mutations in *Kindlin 3*, an integrin cytoplasmic tail binding adaptor that is essential for integrin activation. Patients with LAD-III have similar manifestations as those with LAD-I but with milder symptoms. Unlike LAD-I, increased bleeding tendency is the major source of morbidity. Platelet aggregation requires both β1 and β2 integrin activation, and because of the integrin activation defect in these patients, bleeding severity is increased (48).

Autosomal dominant Hyper IgE syndrome (AD-HIES) is a multi-system disorder characterized by elevated serum levels of IgE, recurrent cutaneous and pulmonary bacterial and fungal infections, development of pneumatoceles, chronic skin dermatitis, and many skeletal and dental abnormalities (49). Staphylococcal infections of the skin and lung are often indolent and lack characteristic inflammatory characteristics (cold abscesses). Loss of function mutations in signal transducer activator of transcription 3 (50) lead to loss in production of Th17 cells and are causative of AD-HIES (51). Neutrophils in patients with AD-HIES have a profound defect in chemotaxis. Diagnosis is based on recognition of the constellation of symptoms along with often profound elevation in serum IgE levels. Treatment consists of antibiotic prophylaxis.

## Disorders of Neutrophil Ingestion and Degranulation Granules

Following phagocytosis, phagosome membranes fuse with neutrophil granules and granular contents are released into the phagosome lumen where direct microbial killing occurs. These microbicidal products are contained within four types of secretory granules: azurophilic (primary), specific (secondary), gelatinase (tertiary), and secretory vesicles (52). Defensins, neutrophil elastase, lactoferrin, and gelatinase are released upon stimulation of the neutrophil from certain infections. Granules can be easily visualized within neutrophils *via* light and electron microscopy.

Chediak–Higashi syndrome (CHS) is an AR disorder caused by defects in *LYST* leading to defects in granule morphogenesis (Figure 1C) with delayed and incomplete degranulation (28, 53). Clinical manifestations include oculocutaneous albinism, neurologic disease, immunodeficiency, and mild bleeding tendency. Natural killer cells are present but function abnormally, as do neutrophils with abnormal chemotaxis and killing both causing an increased risk of bacterial infections. Platelets have irregular morphology; mild bleeding is a common feature of CHS. Neurologic features include cognitive impairment, peripheral neuropathy, ataxia, and parkinsonism. Giant peroxidase positive granules that coalesce azurophilic and specific granules are present within the peripheral neutrophils and are even more prominent within bone marrow-derived neutrophils of CHS patients. Pigment clumping also can be found on hair from CHS patients. About 85% of CHS patients enter the accelerated phase of disease with lymphoproliferative infiltration of the bone marrow and other reticuloendothelial system organs. Treatment consists of chemotherapy followed by HSCT for the accelerated phase (28, 53).

Neutrophil-specific granule deficiency (SGD) is a rare neutrophil defect in which neutrophils lack specific granules and, therefore, have virtually absent lactoferrin production. Clinical manifestations include susceptibility to severe invasive pyogenic infections with *Staphylococcus aureus, Pseudomonas aeruginosa*, and *Candida albicans* (54). Most patients present in the first few years of life with severe infection. SGD is caused by AR mutations in CCAT/enhancer binding protein epsilon (C/EBP-ε) (55). This defect in C/EBP-ε blocks the transition of neutrophil development from the promyelocyte to myelocyte stage. The pathognomonic feature of SGD is a paucity of specific granules and predominantly bilobed nuclei that can be visualized on a peripheral smear (Figure 1B). Neutrophils from SGD patients also show abnormal chemotaxis but with normal aggregation, impaired disaggregation, and decreased bacteriacidal activity (55, 56). Diagnosis of SGD is made by careful examination of a peripheral smear and confirmed with molecular testing. Treatment consists primarily of use of anti-bacterial prophylaxis and possibly HSCT (56).

## Disorders of Neutrophil Killing

Prior to exposure to microbes, the neutrophil NADPH oxidase is inactive with its subunits residing in different cell compartments. Some are membrane bound (gp91^phox^ and p22^phox^) and others are cytoplasmic (p47^phox^, p67^phox^, and p40^phox^). After intracellular ingestion of bacteria and fungi, the components of the NADPH oxidase come together in an oxidative burst shuttling electrons across the phagosomal membrane from cytoplasmic NADPH to molecular oxygen. These reactive oxygen species then directly kill ingested microbes (57).

Mutations in all five structural genes that comprise the NADPH oxidase cause chronic granulomatous disease (CGD) (Table 2) and occurs in approximately 1:200,000 (58). The majority of patients with CGD present before age 5 with a severe or recurrent infections. The skin, lungs, lymph nodes, and liver are the most common sites of infection with a narrow spectrum of catalase-positive organisms. Infections from *Staphylococcus aureus, Burkholderia cepacia, Serratia marcescens, Nocardia* species, and *Aspergillus* species are the most common in North America. Formation of granulomata and a dysregulated inflammatory response to infection are a leading cause of morbidity in CGD patients. Diagnosis of CGD relies on direct measurement of superoxide production; the dihydrorhodamine (DHR) assay is the most commonly used and accepted test to diagnose CGD. The DHR assay uses flow cytometry to measure the production of hydrogen peroxide in the presence of peroxidase and directly correlates with superoxide production by the NADPH oxidase (59). Management of CGD patients relies on life long anti-bacterial and anti-fungal prophylaxis and interferon gamma. Treatment of the immune dysregulation of CGD is often accomplished by the use of corticosteroids or other immunosuppressants. Allogeneic HSCT can cure CGD, and new gene therapy protocols offer a potential cure as well (57).

**Table 2:** Molecular defects of the NADPH oxidase causing CGD (57).

Gene	Protein	Inheritance pattern	Percentage
*CYBA*	p22^phox^	AR	6%
*NCF1*	p47^phox^	AR	20%
*NCF2*	p67^phox^	AR	6%
*NCF4*	p40^phox^	AR	1 individual
*CYBB*	gp91^phox^	XL	70%

*CGD, chronic granulomatous disease; AR, autosomal recessive; XL, X-linked*.

Myeloperoxidase (MPO) deficiency is a common AR disorder caused by mutations in the *MPO* gene. MPO deficiency inhibits formation of hypochlorous acid from chloride and hydrogen peroxide. Despite the significant *in vitro* killing defects, there is a lack of clinical symptoms present in patients with MPO deficiency. No specific treatment, including the use of prophylactic antibiotics, is recommended (60).

Glucose-6-phosphate dehydrogenase (G6PD) catalyzes the two reactions of the hexose monophosphate shunt pathway responsible for forming NADPH. Mutations in G6PD cause a gradual decay in G6PD which have little effect on the short life span of neutrophils. The majority of patients with G6PD deficiency develop red cell hemolysis triggered by oxidative stress. However, a few G6PD mutations have led to very low levels of G6PD leading to severe hemolytic anemia and NADPH oxidase deficiency that clinically resembles CGD (61).

## Conclusion

Across species, neutrophils are critical for host defense against invasive bacteria and fungi. Evolution of neutrophils in humans has developed into an eloquent process of neutrophil ontogeny, trafficking, and killing to become a major first line defense against infection. Defects in neutrophil quantity, adherence, chemotaxis, and killing all lead to severe and potentially life-threatening disease in humans, underscoring the important role of the neutrophil in the immune system. Dissecting the molecular pathology of disorders of neutrophil function has given us unique insight into the primary means by which the innate immune system confronts pathogen challenges. Further investigations of similarities and differences between species in how neutrophils function has considerable potential for revealing the inner workings of a complex mechanism of host defense.

## Author Contributions

JWL developed and wrote this review.

## Conflict of Interest Statement

The author declares that the research was conducted in the absence of any commercial or financial relationships that could be construed as a potential conflict of interest.
